# A typology for digital leadership in higher education: the case of a large-scale mobile technology initiative (using tablets)

**DOI:** 10.1007/s10639-022-11483-w

**Published:** 2022-11-29

**Authors:** Norma Ghamrawi, Rana M. Tamim

**Affiliations:** College of Education, Qatar University, Doha, Qatar

**Keywords:** Digital leadership, Mobile technology, Educational technology, Higher education

## Abstract

This study investigated a digital reform initiative, rated excellent by the government, of one higher education institution (HEI) in an Arab State in the Gulf. The focus of the study was to develop a digital typology, while exploring the leadership attributes that characterized the core leadership team, as they accomplished the migration towards a digital culture in one year, within a context where faculty members showed resistance against digitalization. The study was conducted immediately after the implementation of the initiative that took place over the course of one year, just before the Covid-19 pandemic. Semi-structured interviews were conducted with all the six key individuals in the leadership team who led this initiative. Data was analyzed using content-based analysis. Findings of the study were used to synthesize a 5D typology for digital leadership attributes: (1) Digital competence; (2) Digital culture; (3) Digital Differentiation; (4) Digital governance; and (5) Digital advocacy. The paper provides in depth discussion how these attributes supported the adaptive ability of a Higher Education Institution towards accepting digitalization.

## Introduction

Leadership has long been considered as key for the success of any educational reform (Ghamrawi, 2011) and has been traditionally defined as the ability to affect others to achieve goals (Northouse, 2021). The literature on educational leadership is very rich, including but not limited to: transformational leadership (Burns, 1978), transactional leadership (McCleskey, 2014), inclusive leadership (Northouse, 2021), ethical leadership (Heifetz, 1994), servant leadership (Ghamrawi & Al-Jammal, 2014), and many others.

Among the newly coined leadership terms in the literature is digital leadership, which has been given varied names such as ‘digital technology’, ‘technology leadership’, and ‘e-leadership’ (Bennis, 2013; Oberer and Elkollar, 2018; Ordu & Nayır, 2021; Gfrerer et al., 2021; Mccarthy et al., 2021). Despite being given different names, digital leadership is viewed broadly as a tool that promotes digital learning (the use of digital tools to foster learning) and leverages digital literacy in educational settings (skills needed to succeed in a society where information is accessible through digital technologies such as internet platforms, social media, and mobile devices) (Fareen, 2022; Gfrerer et al., 2021; Mccarthy et al., 2021; Northouse, 2021).

Despite the depth and breadth of research on digital leadership in business, military and other fields (Ehlers, 2020), there is very little research carried out in education (Karakose et al., 2021; Petry 2018), and particularly in higher education (Ehlers, 2020). Studies in K-12 settings have broadly focused on the impact of school leaders’ digital leadership on teachers’ performance during COVID 19 pandemic, such as: (a) examining the digital competencies of school principals through the lens of their teachers (Karakose et al., 2021); (b) exploring the impact of principals’ digital leadership skills on teachers’ digital teaching (Hamzah et al., 2021); and (c) investigating the potential of digital leadership in transforming schools into professional learning communities (Sterrett et al., 2020). On the other hand, the emphasis of the studies carried out in higher education has been on digital education including open universities and online courses (Cunha et al., 2020), knowledge management in digital age (North et al., 2018), and exploring strategies for the digitalization of HEIs.

While the published work on digital leadership broadly focused on the digitalization of schools, none of those explored the leadership attributes initiative leaders, to the best of the knowledge of the researchers. This is, in particular, the gap that this study aimed to address.

### Purpose of the study

This study aimed at developing a typology for the digital leadership attributes that signified individuals in key leadership positions, while leading a large-scale mobile technology initiative, receiving a lot of acknowledgement within the educational community in an Arab State in the Gulf. The study did not focus on the initiative itself, but rather on the attributes exhibited by its leadership team to make it succeed.

#### Context of the study

Upon their introduction to the classroom in 2013 and 2014, tablets were publicized as “smart” classroom technology innovations (Cuban & Jandrić, 2015). Similar to many previous education technology advances, the general assumption was that these devices would shift technology’s role from a delivery tool to an enabler that enriches the learning experience (Tamim et al., 2015a, b). With such a promise, there was a launch of institutional, regional, and national initiatives that made use of mobile devices in K-12 and higher education contexts including laptops, mobiles and tablets (Tamim et al., 2015a; Hollebrands, 2020). With the hype garnered by handheld devices, a number of countries from North and Latin America, Europe, Asia, Africa and the Middle East, as well as the Pacific, embarked on large-scale government-led initiatives to distribute tablet-computing devices to students in educational contexts (Tamim et al., 2015a, b). One of the largest initiatives was implemented by one State in the Gulf, where more than 14,000 tablets were distributed to students in the three Federal universities.

The study at hand attempted to capture digital leadership implications from the implementation of the use of tablets at one of the federal universities, leading to the development of a typology for the digital leadership attributes that leaders exhibited, whilst engaging their target audience in a digital culture (a workplace with increasing reliance on digital tools and technologies).

#### Importance of the study

The digitalization drive of Higher education Institutions (HEIs) is on the rise globally (Xiao, 2019), and is shaping the new reform initiatives in higher education (Fareen, 2022; Williamson & Hogan, 2021; Xiao, 2019). In fact, technology has advanced learning opportunities to a new level (Wasserman & Berkovich, 2022), by supporting ‘personal learning ecosystems’ which are customizable personal digital learning spaces characterized by flexibility and adaptability (Fareen, 2022).

While most studies have explored the interactions among teachers and students, slight attention has been given to factors that support digital reform initiatives in HEIs (Berkovich & Wasserman 2019). As stated earlier, this study aimed at investigating a successful digital reform initiative, rated excellent by the government, of one HEI. The focus was on developing a digital typology while exploring the leadership attributes that characterized the core leadership team as they accomplished the migration towards a digital culture in one year.

Success criteria set by the ministry included the: (1) number of trained students and faculty members; (2) degree digital tools were being used; (3) development of a digital culture within the university; (4) enrichment of the overall quality in the research knowledge in the postgraduate system; (5) the cultivation of the creativity in the course offerings; and (6) the creation of a strong task force that sustain the initiative. The success criteria were converted into 106 measurable Key Performance Indicators (KPIs), which were assessed by external international teams to judge achievement.

Prior to this initiative, the usage of tablets was very limited and not widely used at the level of both instructors and students. Findings of this study are of value for any leadership team in any HEI that is interested in digital reform.

## Literature review

### Challenges confronting higher education

Higher Education Institutions (HIEs) are often criticized for preserving the structures on which they were established in the 11th century and their failure to evolve at the same rate of change impacting societies and the market (Buler, 2014; LeBlanc, 2018; Mehaffy, 2012; Stewart, 2018). In this line, HEIs are also criticized for resisting wider usage of technology including open access resources and distance learning (LeBlanc, 2018). While this at large relates to faculty members’ anxiety from the dominance of technology over their authority which underlies traditional classrooms (Mehaffy, 2012), this comes at a cost on their HEIs because it tends to encourage students to refrain from enrolling in their programs (Hixon et al., 2012; Leal Filho et al., 2020). This is partly because students of the current millennium are more open to technology and look forward for customized learning ecosystems that match their learning profiles (Hixon et al., 2012; LeBlanc, 2018; Mehaffy, 2012).

In this line, several researchers from several parts of the world emphasize the importance of adaptive leadership (Nelson & Squires, 2017; Stewart et al., 2018) which entails the readiness of leaders in HEIs to swiftly confronting challenges, and rendering them as opportunities for their institutions (Thompson & Miller, 2018; Paganelli & Cangemi, 2019).

### Digital leadership

The use of technology has often been perceived as an opportunity to support student-centered classrooms (Montrieux et al., 2015). Research findings in the educational technology field suggest positive impact on students’ performance because it leverages engagement (Dunn & Kennedy, 2019). The perceived benefits of using educational technology can be categorized as either functional or psychological (Zhuang & Xiao, 2018). Functional benefits are defined in terms of how the product or tool helps the users to get the job done, whereas psychological benefits relate to the kinds of positive feelings users have because of the product or tool being used (Hollebrands, 2020). Findings indicate that functional and psychological benefits motivate students to learn and achieve better performance in their courses (Roblin et al., 2018).

Yet, any attempt to introduce technology into educational settings is often linked to people in key leadership positions (Lei, 2010; Shin et al., 2012; Palao et al., 2015; Tamim et al., 2015a; Navarro-Martinez & Peña-Acuña, 2022). Leadership lies at the heart of any educational reform as per research from various parts of the globe (Ghamrawi, 2010,2011,2013; Dakkak, 2011; Harris, 2013; Tabari, 2014; Bush, 2020; Bros & Schechter, 2022; Genelza, 2022). In other words, the success of any reform in education may not be realized without the efficient involvement of leaders. As such, a new form of leadership has been coined in the literature: digital leadership (Ordu et al., 2021).

According to Scheninger (2014), as schools are requested to change in light of 21st century demands, leadership should do the same. The same challenge that confronts both parties, which is technology, prescribes the required change. So, Scheninger (2014) argues that, while schools are called to migrate to the 21st century, parallel to that, leadership needs to voyage towards digital leadership.

When this is the case, it becomes important to distinguish the skillset that characterize digital leaders. According to Eberl and Drews (2021), several terms are used in the literature to indicate digital leadership. In fact, based on an extensive review of the literature, Mccarthy et al. (2021) suggested that they got more confused with the definition upon reviewing 87 research articles. They arrived at a bunch of terminologies describing the term, including: ‘digital strategist, digital culturalist, digital architect, customer centrist, organisational agilist, data advocate, business process optimiser and digital workplace landscaper’ (Mccarthy et al., 2021, p. 1).

In his attempt to describe digital leadership, Scheninger (2014) proposes five elements that describe effective digital leadership: (1) visionary leadership, (2) learning culture in the digital age, (3) excellence in professional practices, (4) systemic improvement, and (5) digital citizenship. Moreover, Panshin et al., (2019) describe digital leaders as continuous learners who endlessly seek intellectual curiosity and new knowledge. They get involved in the application of techniques to use digital data to achieve goals (Antonopoulou et al., 2020). In the same vein, Richardson et al. (2012) suggest that digital leadership is key in improving the repertoire of teaching practices for teachers. This is in line with Chang’s (2012) study, which confirmed a direct relationship between digital leadership capacity and K-12 teaching effectiveness.

Alternatively, some terms associated with digital leadership in the literature include digital thinking (Peng, 2021), digital competence (Schiuma et al., 2021), skillfulness in using ICTs (Antonopoulou, 2020), the creation of digital cultures (Mihardjo et al., 2019), social influence (Stana et al., 2018), innovation (Tanniru, 2018), team-orientation (Oberer & Erkollar, 2018), digital transformation management (Zhong, 2017), behavioral digitization (El Sawy, 2016), and organizational digitization (Larjovuori, 2016).

The illustration of each of the above terms is provided in Table 1.

**Table 1:** Illustration of Common Terms Describing Digital Leadership in the Literature

Term	Researcher(s)	Illustration
Digital thinking	Peng (2021)	Set of skills needed to transform real-life challenges to problems that can be handled using technology.
Digital competence	Schiuma et al., (2021)	Confident and critical use of technology in work, leisure and communication.
ICT skillfulness	Antonopoulou (2020)	Professional use of technology in everyday life.
Digital culture	Mihardjo et al. (2019)	A workplace with increasing reliance on digital tools and technologies.
Social influence	Stana et al., (2018)	Using technology to create an impact on social media.
Innovation	Tanniru (2018)	The skillset that leads to the development of new technologies.
Team-orientation	Oberer & Erkollar (2018)	The skillset fostering collective effort rather than individual task completion.
Digital transformation management	Zhong (2017)	Cultural change making organizations continually challenge their status quo to operate at higher levels of quality.
Behavioral digitization	El Sawy (2016)	Understanding behaviors of users through their digital input to amend business strategies.
Organizational digitization	Larjovuori (2016)	The process of embracing technology by all members of an organization.

At this point, digital leadership may be better understood by reverting to the basic definition of leadership endorsed by most researchers within the literature of educational leadership. In fact, leadership has been defined as the act of motivating members of a community to achieve a desired goal (Al-Jammal & Ghamrawi, 2013). In the case of digital leadership, the goal is technology endorsement and usage: making advantage of its powerful aspects in learning and teaching as well as management of learning (Palao et al., 2015; Navarro-Martinez & Peña-Acuña, 2022).

### Synthesizing a definition for digital leadership

Digital technology became an integral element of higher education, impacting all aspects of student learning (Selwyn, 2016; Henderson et al., 2017; Barak, 2018). This includes promoting global citizenship (Choi et al., 2017; Redecker, 2017; Nikou & Economides, 2018), fostering collaboration (Sosa Neira et al., 2017; Oliver & de St Jorre, 2018), social networking (Schindler et al., 2017; Redmond et al., 2018), supporting student-teacher communication (Atmacasoy & Aksu, 2018), and improving student self-efficacy (Alioon & Delialioğlu, 2017). At the same time, some of the literature on educational technology remains cautious, considering technology, like any other tool in education, would fail to promote improved learning experiences to learners unless used wisely and effectively by effective users (Tamim et al., 2011; Popenici, 2013; OECD, 2015; Englund et al., 2017).

While the landscape of educational technology research is already complex, the introduction of the concept of leadership made it never less intricate. This is manifested by the literature already discussed earlier in this paper, showing diverse understanding for digital leadership (Cunha et al., 2020; North et al., 2018; Sterrett et al., 2020; Karakose et al., 2021; Hamzah et al., 2021).

If leadership is all about motivating others to fulfill certain goals (Bennis, 2013), then digital leadership is all about influencing subordinates to use digital assets in order to achieve organizational goals. Moreover, if a transformational leader pursuits the potential motives in followers, to incite their full engagement in establishing organizational goals (Scuotto et al., 2022), then a digital leader is a transformational leader who seeks digital transformation of his/her organization through capitalizing on the best interests of their employees.

As such, it may be argued that digital leadership is an evolved form of transformational leadership; one that is centered on supporting the organization to respond effectively and diligently to the rapidly changing technology through innovative solutions to ensure they remain situated ahead of the curve and thus remain competitive.

While different researchers in different fields define digital leadership differently, this study adopts the conception of digital leadership endorsed in educational research as being the set of attributes and competencies that those in leadership positions adopt and utilize, in order to secure a digital culture within their communities. Within those communities, members embrace digital technologies and make use of them, in their strive to learn and support the learning of others.

Given the above definition of digital leadership, this study attempted to investigate the attributes of digital leaders that support them in the digitalization initiatives. It thus serves those interested in the leadership dimension that supports the promotion of educational technology in educational settings; yet at the same time, adding to the literature a typology for better understanding of the attributes and factors of the concept.

## Research methodology

### Case study

This study was based on the interpretive paradigm, seeking data to earn deep empathetic understanding of the researched topic through qualitative interviewing. It utilized the case study methodology to arrive at multi-faceted understanding of complex issues in their real-life context (Cresswell 2013). The researchers’ approach to case study methodology is in line with that of Stake’s (1995), who suggests that case studies are epistemologically built on constructivism and existentialism. As such, he asserts that researchers carrying out case studies construct knowledge, rather than discovering it. As such, according to Stake (1995), researchers interpret or gather interpretations of the constructed knowledge during the course of their exploration. With this in mind, readers themselves can carry out personal interpretations of this knowledge, according to Stake (1995), as he acknowledges the existence of ‘multiple perspectives’ for a given case.

### Study context

All the three HIEs that implemented this initiative were contacted by the lead researcher for conducting the research study. The research team (who were externals to all contacted HEIs) made a prior agreement that the first one to respond positively would constitute the study sample. None of the other two ever responded to the research invitation. As such, this HEI was selected to take part in the study, and no follow up with the other two HEIs was made.

After receiving ethical clearance from the research office of this HEI, potential participants were approached for their consent to be interviewed. All the six key members of the management team, namely two senior administrators, two senior IT personnel, and two frontrunner faculty members known as iChampions, agreed to participate in the study.

As such, the sample of this study included the whole population of educational leaders who were involved in a large-scale mobile technology initiative. All six participants constituted the task force that led this initiative, and were in direct contact with the University President Office. Each member was leading a sub-team in one of the faculties of the university.

The interviews were conducted individually with each participant, face-to-face on campus, by two of the researchers and typically lasted around 30 min each. As requested by the university, participants were sent the interview schedule alongside the invitation to participate in the study, so they knew ahead of time what data the researchers were seeking.

The interviews focused on exploring the attributes that supported the success of the initiative, as well as the resistance that confronted them. In fact, the literature suggests that reform in education is always confronted with resistance (Molla & Cuthbert, 2022; Price & Regehr, 2022). Members of a community tend to oppose changing their routines, as this pushes them out of their comfort zones. Particularly with technology, faculty members are more prone to rebut change (Habib et al., 2021), as they fear they could lose authority and control (Mpungose, 2020).

At the beginning of the interview, the researchers introduced themselves, and communicated with full transparency the purpose of the study, how data will be used and their right to withdraw at any time. One of the researchers led the session and the other wrote notes, and was there for data corroboration later on, ensuring accuracy of captured data. Questions were posed and participants were left to answer with no disruption. At this stage, the interviewer would ask additional questions to elicit better understanding of the answer and hence earn a comprehensive understanding of the response. When done, the lead researcher recapitulated the answer to ensure validity. The interviews were recorded and transcribed later on word-by-word, except for one case where the participant did not feel comfortable with recording, and as such, notes were taken by both researchers and compared later on for accuracy.

The explored model was adopted by all the three universities where the tablet initiative was implemented, as it was developed by a task force. This task force held three meetings throughout the year during which the initiative was implemented. It involved a total of 13 members: a government representative, presidents of the three universities, their iChampions and one IT personnel.

All participants were long-standing personnel members at the University. The two senior administrators, were leading figures in the initiative, and were involved from the early stages of discussion between the university and government financial representatives who audited the budget of the project. One was a senior administrator who was involved in the conceptualization of the project at the university level, while the other was working in a managerial capacity to support the implementation aspects of the initiative. The two senior IT personnel were instrumental in the implementation of the initiative. One was a lead IT manager for the project with the second being a lead technical support member responsible for the technical training of students and faculty members.

Finally, the faculty members who participated in the interviews were members of the iChampions team who were described earlier in this paper. They were provided with focused professional development relating to tablet integration for student-centered learning. In addition, they were given course release time enabling them to serve as mentors to other faculty members.

### Ethical considerations

The interviewed team was only involved in the realization of the milestones of the initiative. On the other hand, the governmental representative carried out the role of a financial audit, ensuring the budget was being spent according to the initiative’s masterplan. None of those was involved in judging the quality of the initiative, a task that was left out to an external international team who visited universities at its completion for an ‘inspective’ evaluation of the achievement of the HEI against the Key Performance Indicators (KPIs) set by the Ministry. This team consisted of experts in digitization, education, and quality assurance in HEIs, bearing international expertise with international accrediting bodies, and international development organizations.

Coupling this non-evaluative role of interviewees on one hand, with the nature of the interview questions posed on them, on the other hand, tend to support the belief that those interviewees were free to express their thoughts freely.

### The interview guide

The research instrument consisted of an interview schedule with the following two questions:

1. What attributes do you think contributed to the success of the initiative?
2. How did you overcome the resistance that confronted you while leading the initiative?

### Data analysis

Due to the small sample size, no qualitative data analysis software was required. This decision was further supported by the authors’ need to develop a deep understanding of the data at hand. Consequently, manual analysis was used as suggested by Creswell & Clark (2017). The process of data analysis followed a content analysis approach (Boyatzis, 1998), where the data was independently coded and labeled by two researchers to identify and organize codes and meanings. A meeting between the researchers followed initial coding to compare and discuss the codes and resolve conflicts and decide on the common classification and main themes (Creswell & Clark, 2017).

### Attributes that contributed to the realization of the initiative

Addressing the aspects that supported the realization of the initiative, participants’ responses were classified under three main themes as presented in Table 2.

**Table 2:** Emerging themes-personal attributes contributing to the realization of the initiative

Theme	Illustration	Terminology used by researchers
Confidence in using technology	*Leaders of technological initiatives should be experts themselves in terms of using technology.*	Digital Literacy
Establishing the culture	*Leaders manage success on technology initiatives through the establishment of a community who share the same digital norms, rituals, language and symbols.*	Digital Culture
Securing personalized learning opportunities	*Leaders of technological initiatives should focus on personalizing learning opportunities for learners, and make that explicitly. This is considered a buy-in for end-users.*	Digital Differentiation

Each of the three emerging themes, digital leadership, digital culture and digital differentiation, are addressed in what follows.

### Digital literacy

Participants in the study all mentioned the importance of being confident in using technology. By this, they meant that leaders of technological initiatives should be technologically expert themselves in order to be able to promote it effectively and disseminate it within their communities.

“I think that by far, the most important attribute that allowed me to succeed in my task had to do with my ability to use tablets professionally. If I were incompetent in using tablets, I do not think I would have been able to achieve well” (P2).

“My excellent background and experience in using technology was the most critical element for my success along other team members. You need to know your product very well if you ever want to sell it “ (P1).

So, there is a convergence at the theme that the success of this initiative relies heavily on the know-how of technology and its tools. Marketing a product, entails the importance of knowing the features and details of that product.

#### Digital culture

Another emerging theme from interviews was the ability of the leader to develop common norms, rituals, language and symbols for community members. In other words, the leader should be involved in the creation of a culture where members share basic common components. This was a point made by the majority of interviewees.

“ I believe that one of the things that boosted our work was the set of meetings we held at all levels at the very beginning of the initiative. During those meetings, we focused on speaking the same language when dealing with tablets. There so many things we discussed prior to launch. So, if you read comments, tweets, posts, etc… of members of our community, you would tend to think that those people are natives of the same country. This itself created a kind of self-belonging for members and helped us as leaders to mobilize them towards the desired goals” (P4).

“I have worked on several projects related to technology, but honestly this one was the most successful and one of the reasons had to do with the development of common language and attitudes towards technology before the inception of the initiative” (P5).

That is to say, leaders of technological initiatives should be involved in the development of a culture conducive for accepting and showing pride in technology.

Finally, leaders should aim at the creation of cultures that cements members to common beliefs, attitudes, and language.

“......so I can say that people started with full resistance, then moved to a state where they thought the initiative was useful but their plates were too loaded to take it, then moved to s state where they thought it was too useful to be missed. This was definitely, because they saw us endorsing and using it. We did not limit ourselves to the generation of policies but managed to address what kind of behaviors was expected from our faculty members and celebrated problem identification and suggestions of solutions by faculty” (P5).

#### Digital differentiation

Acknowledging personalized learning spaces and learning according to ones needs, interests and availability was another attribute underscored by the interviewees. All participants thought this was one of the buy-ins that they managed to deploy for attracting participants.

“ One of the things that supported me in attracting learners was personalization of learning opportunities. I think this is probably the most important element that I believe could be used to attract learners. When you tell students that courses through tablets can be taken based on your level, abilities, strengths and time, they tend to be more interested in what you are advocating for” (P. 6)

“I think that one of the roles that I would emphasize if I am to run another large scale project is marketing individualization. By individualization, I mean that letting students know that technology allows them to learn via so many doors and not only one door. They can decide when and where to take up a lesson. They can also decide to match what they are taking based on their backgrounds and interests” (P. 3).

Thus, a third digital leadership attribute recommended by participants relates to differentiation of learning opportunities technology offers. It seems that both students and faculty appreciated the customized and personalized learning opportunities that digital technology offered them. However, while technology seems to offer this opportunity, it is the role of the leader to market this feature as part of his endeavors towards managing the success of digital initiatives.

### Overcoming resistance against technology initiative

Themes from interviews pertaining the aspects that supported the leads of the tablet initiative harness the resistance of community members against the tablet initiative are presented in Table 3.

**Table 3:** Emerging themes –Overcoming resistance confronting the initiative

Theme	Illustration	Terminology used by researchers
The availability of an overarching authority	*Leaders of technological innovations benefit from top-down authority in terms of decision-making.*	Digital Governance
Marketing ideas at all levels	*Leaders of technological innovations should be marketing their ideas across their initiative’s path.*	Digital Advocacy

Each of the two emerging themes, digital governance, and digital advocacy, are addressed in what follows.

### Digital governance

Contrary to expectations, all participants suggested that the presence of a higher authority (which is the university board) making decisions made it simpler for them to lead the project. They explained that this minimized controversial discussions on steps to be taken, and how they were supposed to be handled.

“The role of the board as an overarching body played a positive role in handling resistance of professors in particular. When confronted with divisive opinions, we used to tell professors that decisions were board-related. Honestly, this cut down a lot of the back and forth discussions that normally take place and often hinder progress.” (P. 4).

“ You might say I am an authoritarian leader, but trust I am not. Ask about me. I am saying this before hand because what I will tell you now might frustrate you. I think strict decisions coming from above [the board] saved our necks with professors. Anytime they wanted to argue, we would tell them check with the board. They would never check with the board and decide to flow with the mainstream. ” (P. 2).

Thus, it can be argued that the presence of an overarching governing body within technology initiatives could support leaders in terms of minimizing disruption resulting from conflicting opinions pertaining them. It might be thought that the top-down leadership model prevails in the context where the study was conducted, however, as some of the quotations from participants show, they were aware of leadership styles, and asserted they were non-authoritarian themselves.

### Digital advocacy

A final attribute that technology leaders should exhibit whilst leading digital initiatives is advocacy. This is as per majority of the participants who accentuated the value of marketing ideas, prior and during their launch. This attribute seems to support leaders in better addressing resistance.

“I think that my very best advice for leaders of digital initiatives is to review business marketing [laughs]. You should plan for marketing what you intend to do before, during and after you do it. Stress the benefits various parties would gain. Focus on the bright side of tasks. Keep on talking before walking.” (P. 1).

“ I focused a lot on highlighting personal gains for professors to buy them into the initiative. I think this is so crucial. It felt like being a non-stop TV add advertising current and next steps. I put the maximum effort on young faculty members whom you are strong believers of technology. These really make a big difference” (P. 5).

In other words, leaders of digital initiatives seem to be better supported when they consider marketing their ideas. Advocating the initiative, its elements or paths seem to impact positively on community members, who tend to find a rationale for getting on board.

### A typology of the digital leadership

**Fig. 1:**
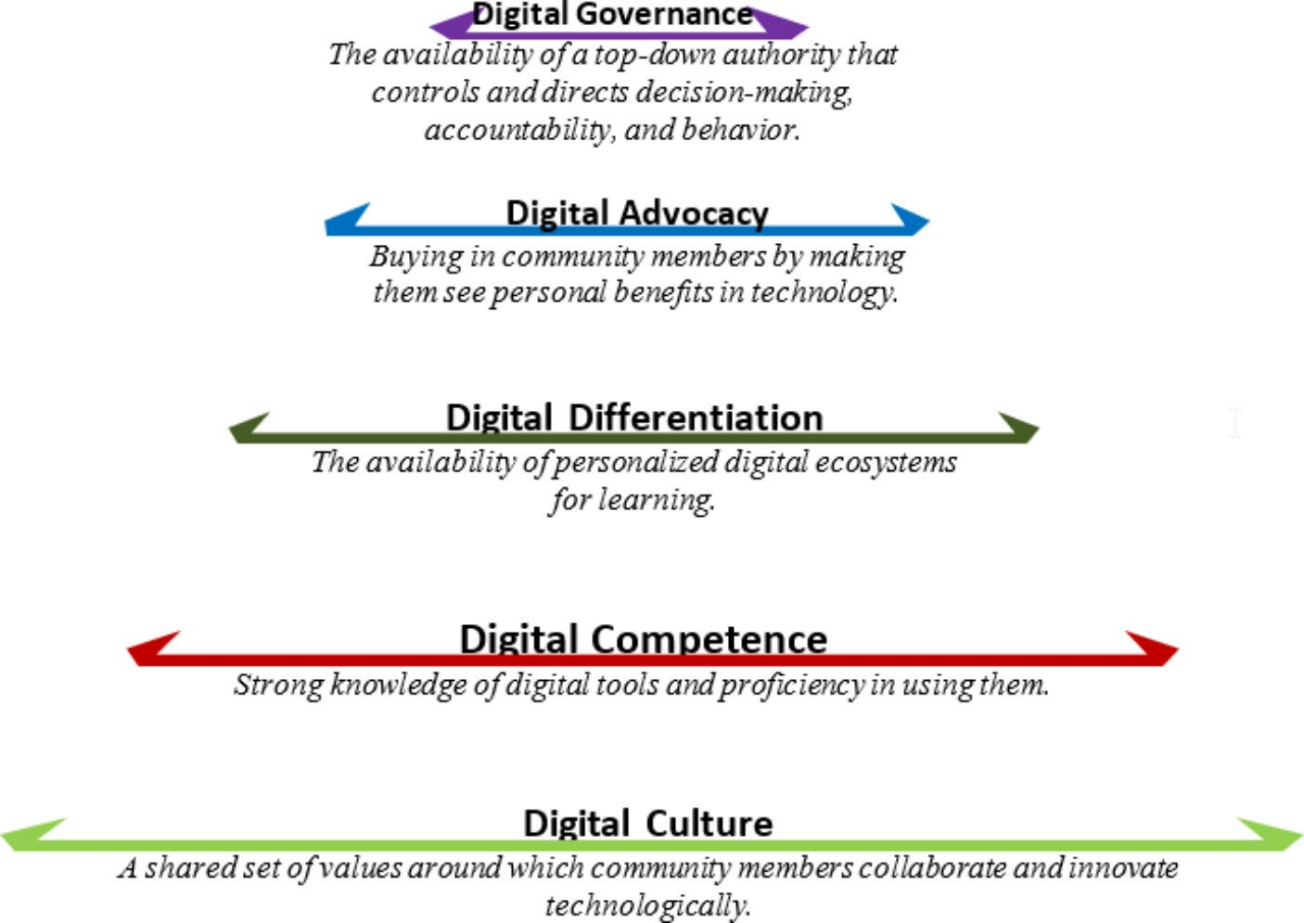
A 5D Typology for Digital Leadership

The typology of digital leadership attributes that is derived from this study is presented in Fig. 1.

The 5D typology for digital leadership, which has been synthesized using the responses of interviewees, provide a visual picture of how leaders within the context and scope of this large-scale technology initiative supported and nurtured their digital initiatives.

First, digital leadership entails digital competency through which leaders are expected to exhibit strong knowledge of digital technologies. Leaders should be highly proficient themselves in dealing with such technologies. In other words, they should be able to exhibit proficiency in using digital tools they aim to promote to their communities. This finding comes in line with the literature on digital leadership (Scheninger, 2014; Zhong 2017; Mercader; 2020; Ehlers, 2022), suggesting that a digital leader needs to be quite competent in technology in order to promote it effectively into the wider community of the educational organization.

It may be concluded that, when it comes to technology, strategies, plans, and process can go down the drain, if faculty members do not buy-in into the new way of thinking. This is a tricky part of contributing to the success of any reform initiative in general, and digital technology in particular. Subordinates tend to be influenced positively when they see their leaders ‘walking the talk’ of digital technology, rather than ‘talking the walk’ only. Education leaders would be sending a powerful message to their educational community, suggesting that ‘if I can do it, then you can do it too’.

Secondly, digital leadership entails the development of a digital culture, which is conducive to collaboration and innovation technologically by community members under a shared set of values. Parallel to Scheninger (2014), community members should be able to build alliances centered on student learning and their learning outcomes. Moreover, finding come in line with several studies cited in the literature(England, 2018; Mercader, 2020; Barnes & Gearin, 2022), who stress the role of digital leaders as cultural crafters. Moreover, it is crucial to construct a digital culture, based on a strongly held and widely shared set of beliefs that are supported by strategy and structure. A strongly crafted culture supports the educational community in moving from a stage where they ‘oppose’ the initiative (initial rejection of the initiative), to another where they ‘crib’ the initiative (valuing the initiative but believing they are overloaded), arriving at a stage where they would be open to ‘learn’ (full acceptance of the initiative).

In the same vein, organizations do use formal levers to manage change and promote new organizational structures, roles, and responsibilities, through the creation of policies and the process for monitoring and sustaining them. However, informal levers remain fundamental to encourage faculty to change the way they think, feel, and behave, in order to embed the digital route of doing their day-to-day tasks. Developing policies without targeting critical behaviors may put the transformation at the stake.

A digital culture that is conducive for reform, should be inclusive, solution driven, built on the foundation of knowledge sharing, and accepting to mistakes. Faculty members may never learn unless they take risks. While doing so, faculty do not only identify problems, but also get empowered to come up with possible solutions. Such a culture is the biggest enabler of growth, and the strongest catalyst for change. This is a finding that comes similar to the literature on digital leadership (Habib et al., 2021; AlAjmi, 2022; Carvalho, 2022), which underscore the high impact of positive and inclusive cultures on digital transformation.

Thirdly, digital leaders should emphasize how technology differentiates learning opportunities for its users. This study have shown that making the community aware of the differentiated learning opportunities that new technologies offered them, was integral in the acquiescent of such tools. While the literature emphasizes technology as means for differentiation (Tamim et al., 2015b, 2017), none of those studies have emphasized this feature, as one key feature that digital leaders may use in order to advocate for, when handling new initiatives.

In fact, digital differentiation is probably the most attractive element of digital technology. Differentiation is one of the key challenges for diligent faculty who are keen to address individual differences amongst their students, and responding to the increasingly diverse student population. Digital reformers should take the time and make the right effort to show how technology supports them in engaging their students in different modalities. They should allow them to visualize how their endeavors would be enhanced through technologies, that enable them to vary the rate of instruction, complexity levels, and the various techniques and strategies that supports them in both engaging and challenging students. In other words, digitization should be promoted, especially for faculty members, as an approach for making them work ‘smarter’, rather than ‘harder’, when it comes to responding to students’ diverse needs.

Moreover, digital leadership necessitates digital governance via which community members are directed and controlled under a top-down authority. Such an authority could push them to work towards certain goals with minimal resistance and less controversy. In other words, authority, direction and control are, as equal as, a collaborative culture is, when it comes to meeting objectives and deliver in accordance to standards. This finding came opposite to all findings in the literature, which emphasize the importance of shared decision-making and distributing leadership (Hallinger & Heck, 2009; Ghamrawi, 2013; Harris, 2013; Wenner & Campbell, 2017; Williams & Young, 2022).

One possible explanation can be made in relation to organizational power politics, which according to Alapo (2018) permeates all actions within an organization. Power within an organization does not always connote with authoritarianism as per the social psychologists French and Raven (1959, cited in Alapo, 2018), who distinguished five sources of power: reward, legitimate, expert, referent, and coercive.

While this finding requires further investigation in future research, it may be argued at this point that the kind of power involved in supporting the initiative is legitimate power, which is influence based on position. There have been no mentioning of influence based on rewards (reward power), threatening and punishment (coercive power), influence based on knowledge and information (expert power), nor influence based on charisma and trust (referent power).

Furthermore, digital leadership demands digital advocacy on behalf of the leader through which he/she galvanizes people towards technology usage. Digital advocacy is strongly based on effective communication and supports the digital leader in working towards making community members view their personal benefits and gains, when adopting digital tools. This comes parallel to the literature, which attributes common failure of digital technology initiatives to missed advocacy efforts (Seale et al., 2015; Alasmari & Zhang, 2019; Bolinas, 2022).

Advocating for technology continuously, is a key ingredient for supporting digitization and the creation of a digital culture. While there are so many ways to advocate for technology within an organization, this study suggested capitalizing on its young enthusiastic digital savvy who tend to influence its traditionalists slowly and gradually, yet impeccably. Those members were instrumental in serving as advocates and change agents who encouraged technology adoption through the successes they achieved.

Finally, it should be noted that the 5D digital typology is presented using a pyramid suggesting both ‘hierarchy’ and ‘size’ for each of its elements. In terms of hierarchy, digital governance comes at the top of this pyramid, yet its size is least; meaning that it is a critical element in the digitization process but the presence of a single board or reference is enough. Contrary to this, digital culture, which comes at its bottom is needed largely and widely to support the digital transformation initiative. Each member of the organization needs to be addressed, that is why it is given a large size; however, as a standalone element it has the least effect on supporting initiatives.

## Conclusion

This study aimed at arriving at a typology for digital leadership through an investigation into how key leaders, in one federal university in one Arab State in the Gulf, managed to succeed in expanding the usage of tablets as a learning tool. Findings suggest a typology of five key digital leadership attributes that support leaders of technological initiatives in transforming their communities into digital ones: (1) Digital competence; (2) Digital culture; (3) Digital Differentiation; (4) Digital governance; and (5) Digital advocacy.

Digital leadership relies heavily on the expert knowledge of leaders in position. They should be able to use effectively any digital technologies they aim at expanding within their contexts. They should create a positive culture around shared values of student learning. Through this, digital leaders ensure that members can work and collaborate under the same vision, leading to the outcomes, which constitute the key performance indicators of the project. Yet, this positive environment needs to be further pushed towards achieving its goals, through a governing body that secures accountability, liability and responsibility. Finally, digital leaders should market their ideas and the digital technologies they are after. Through advocacy, leaders serve as eye openers for community members towards the benefits they would make out of espousing the desired digital tools, one of which is the differentiated learning opportunities they secure.

## Limitations

Organizational power politics is a key ubiquitous component of any organization. This study examined leadership aspects that promoted the success of a digital initiative without any focus on organizational power and its politics. It was very interesting to see this as an emergent finding, despite the fact that addressing organizational power within the interview schedule was not intended. However, the study would have been enriched, if this dimension was addressed as part of the interview schedule, since it would allow for a deeper understanding of how power impacts leadership. As such, greater lessons may be learned out of this university’s experience with education technology, by examining the organizational politics, focusing on the antecedents, consequences, and moderating factors, that were conducive for its success.

In addition, it may be argued that interviewees could not speak freely because, as described in the methodology section, two of them were in direct contact with the University President office (UPO), and that a governmental representative was present in general board meetings. It is thus important here to reiterate that members of the interviewed team were not involved in any evaluative or judgmental role pertaining the initiative. The two members who were in contact with the UPO reported on milestones achieved through a digital management system, such as reporting on the number of digitized courses, or the number of trained faculty and staff, at the end of each month. Besides, the governmental representative carried out the role of the financial audit who scarcely met the team for following up on the budget only. A final point to mention here is that the nature of the questions posed on participants supported interviewee-interviewer trust, because they merely focused on personal experiences and opinions.

On the other hand, it may be argued that being a case study, this study does not contribute to the literature of digital leadership. Contrary to this, the literature of case studies by prominent researchers (Robert Yin, Sharan Merriam, Robert Stake) suggest that the case study methodology may be used to generate theories (Yazan, 2015). Siggelkow (2007, cited in Yazan, 2015) argues that even a single case study may be powerful enough to contest a widely held view. In fact, the story illustrated through this case study could be applicable in other contexts as well, as it addressed expanding the usage of technology and digitizing courses in a higher education context. An in-house team led the initiative, working with students and faculty. There might be many HEIs who are interested in a similar initiative, and the typology arrived at could be very helpful for them in their endeavors.

## Data Availability

Anonymized transcripts of interview data are saved by the PI and are available upon request.

## Abbreviations

HEIs: Higher Education Institutions
DL: Digital Leadership
